# Temporal trend of age at menarche in Korean females born between 1927 and 2004: a population-based study

**DOI:** 10.3389/fendo.2024.1399984

**Published:** 2024-06-04

**Authors:** Da Hye Lee, Jaehyun Kim, Hwa Young Kim

**Affiliations:** ^1^ Department of Pediatrics, Chung-Ang University Hospital, Seoul, Republic of Korea; ^2^ Department of Pediatrics, Seoul National University Bundang Hospital, Seongnam, Republic of Korea; ^3^ Department of Pediatrics, College of Medicine, Seoul National University, Seoul, Republic of Korea

**Keywords:** age at menarche, epidemiology, obesity, adolescent, Korea

## Abstract

**Backgrounds:**

The age at menarche has decreased worldwide. Previous studies on Korean adolescents have reported a downward trend in age at menarche. This study aimed to investigate the current trends in age at menarche among Korean adolescents using nationally representative data.

**Materials and methods:**

The study used data from the Korea National Health and Nutrition Examination Survey 2007–2021. A total of 50,730 females born between 1927 and 2004 with information on age at menarche were included. The trend in age at menarche was analyzed according to 15 birth-year groups (with 5-year intervals) using quantile regression analysis.

**Results:**

The mean age at menarche decreased from 16.92 ± 0.06 years for females born before 1935 to 12.45 ± 0.04 years for females born between 2000 and 2004 (*p* <.001). According to the percentile group of age at menarche, mean menarche age decreased by –0.071 years per year (95% confidence interval [CI], –0.072 to –0.070) in total, –0.050 years per year (95% CI, –0.052 to –0.048) in the 3rd percentile group, –0.088 years per year (95% CI, –0.091 to –0.085) in the 97th percentile group (*p* <.001 for all). A decreasing trend of age at menarche was more prominent in the obesity group (–0.080 years per year, 95% CI, –0.082 to –0.078) compared to the non-obesity group (–0.069 years per year, 95% CI, –0.071 to –0.068) (*p* <.001 for both).

**Conclusion:**

Ongoing downward trend in age at menarche was observed in Korean females born until 2004, decreasing by 0.71 years per decade. The downward trend was faster in individuals with a higher percentile of age at menarche and in those with obesity.

## 1 Introduction

Age at menarche has significant health implications. Early menarche is linked to an increased risk of breast cancer, depression, type 2 diabetes, and overall mortality (1–5). Age at menarche can be affected by various factors, including genetics, the escalating global obesity epidemic, and the pervasive presence of endocrine disruptors (6, 7). Numerous have established a correlation between obesity and an increased risk of early menarche (8–10). A systematic review of 17 longitudinal studies indicated that a higher increase in body mass index (BMI) or body weight during childhood could increase the risk of early menarche (9, 11).

Age at menarche has decreased since the mid-19th century in Western countries; however, this trend has varied between countries since the 1950s (12–15). While continuous declines in the age at menarche have been observed in many countries, the decline has slowed down in countries such as the USA and Italy (6, 16–18). In addition, the trend of age at menarche showed a U-shaped pattern in Norway, with an initial decrease in age at menarche in females born between the 1930s and the 1950s, followed by a slight increase in those born in the 1960s (19). In Asian countries, a consistent decrease in the age at menarche has been reported in Japanese females born between 1973 and 2004 and in Chinese females born between 1930 and 1985 (20, 21).

Consistent with the global trend, a downward trend in the age at menarche has been reported in Korea, with a significant decrease noted among individuals born between 1904 and 1994 (22). Research focusing on females aged 12–18 years born between 1988 and 2003, further corroborates this downward trajectory (23). However, recent data regarding age at menarche among Korean females are lacking. Furthermore, it remains uncertain whether this trend varies according to percentile groups of age at menarche. This study aimed to investigate current trends in age at menarche among Korean females, while also investigating the potential differences based on percentile groups and obesity status, utilizing nationally representative data.

## 2 Materials and methods

### 2.1 Study population

This study used data from the Korea National Health and Nutrition Examination Survey (KNHANES) IV–VIII (2007–2021). The KNHANES is an ongoing nationwide cross-sectional survey that employs a stratified, multistage, clustered probability sampling approach to select a representative sample of non-institutionalized individuals residing in Korea. Detailed information on the KNHANES was released by the Korea Centers for Disease Control and Prevention (24). Among the 65,419 female participants from the 2007–2021 surveys, individuals without a recorded age at menarche (n = 14,219) and those born after 2005 (n = 47), potentially at pre-menarche were excluded. In total, 50,730 female participants born between 1927 and 2004 were included. An additional analysis stratified by obesity status excluded 1,561 individuals without BMI information, resulting in 49,169 participants. All participants provided written informed consent before the survey. The Institutional Review Board of the Korea Centers for Disease Control and Prevention approved the use of these data.

### 2.2 Measurements

Participants were categorized into 15 groups based on their birth-year (from 1927 to 2004): before 1935, and at 5-year intervals thereafter. Each birth-year group was subdivided into seven categories according to the percentiles of age at menarche (3rd, 10th, 25th, 50th, 75th, 90th and 97th percentiles). Age at menarche was ascertained through self-reporting in response to an open-ended question in the survey questionnaire: “At what age did you have your first menstrual period (menarche)?” Height was measured using a stadiometer (Seca 225; Seca, Hamburg, Germany), and weight was determined using an electronic balance (GL-6000-20; G-tech, Seoul, Korea). BMI was calculated as weight divided by the height squared (kg/m^2^). Obesity was defined as a BMI of ≥95th percentile for age and sex for individuals under 18 years of age, and a BMI ≥25 kg/m^2^ for those aged 19 years and older, in accordance with the guidelines recommended by the WHO for the Asia-Pacific region and the Korean Society for the Study of Obesity (25–27). BMI measurements taken at the time of the survey were utilized, supported by evidence indicating that obesity in childhood can predispose individuals to obesity in adulthood (28, 29). Socio-demographic characteristics, including obesity status, are presented in **Supplementary Table 1**.

### 2.3 Statistical analysis

Stata 16.1 software (StataCorp LP, College Station, TX, USA) was used to perform all statistical analyses. All analyses were conducted using sampling weights to present estimates representative of the Korean population. The age at menarche was surveyed in whole years; for instance, an age of 15 years included ages from 15.00 to 15.99 years. Therefore, for statistical analysis, the representative age was calculated by adding 0.5 to the surveyed age to reflect the range covered by each reported age accurately. Quadratic regression analyses were used to assess the temporal trends in age at menarche as a continuous variable. The trend in age at menarche was analyzed according to 15 birth-year groups (with 5-year intervals) using quantile regression analysis. A two-sided *p* value of <.05 was considered statistically significant.

## 3 Results

### 3.1 Trends in age at menarche according to birth-year

**Figure 1:**
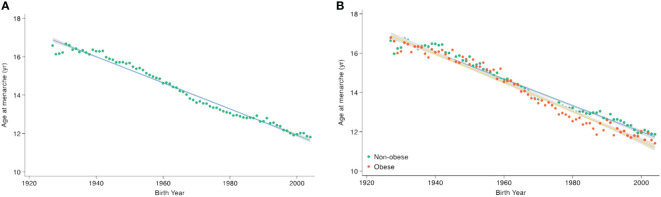
Secular trends in age at menarche according to birth-year. Points indicate the mean age at menarche per birth-year, with trend lines derived using quadratic regression. The gray area indicates the 95% confidence interval. **(A)** Across the total population, **(B)** by obesity status.

**Table 1** shows the age at menarche across birth-year groups at 5-year intervals. The mean age at menarche has declined as the birth-year increases, from 16.92 ± 0.06 years in females born before 1935 to 12.45 ± 0.04 years in those born between 2000 and 2004 (*p* <.001) (**Figure 1A**). When stratified by obesity status, the mean age at menarche ranges from 16.97 ± 0.08 years to 12.48 ± 0.04 years in the non-obesity group, while in the obesity group, there was a decrease from 16.80 ± 0.09 years to 12.25 ± 0.09 years during the entire period analyzed (**Supplementary Table 2**). The overall decreasing trend in age at menarche across birth-years was more prominent in females with obesity than in those without obesity (**Figure 1B**).

**Table 1:** Trends in age at menarche among Korean females born between 1927 and 2004.

			Percentile group of age at menarche						
Birth-year	N	Mean ± SE	3rd	10th	25th	50th	75th	90th	97th
<1935	1,534	16.92 ± 0.06	13.5	14.5	15.5	16.5	18.5	19.5	20.5
1935–39	2,717	16.76 ± 0.05	13.5	14.5	15.5	16.5	18.5	18.5	20.5
1940–44	3,506	16.66 ± 0.04	13.5	14.5	15.5	16.5	18.5	19.5	20.5
1945–49	3,623	16.22 ± 0.04	12.5	13.5	14.5	16.5	17.5	18.5	19.5
1950–54	3,826	15.94 ± 0.04	12.5	13.5	14.5	15.5	17.5	18.5	19.5
1955–59	4,848	15.48 ± 0.03	12.5	13.5	14.5	15.5	16.5	17.5	19.5
1960–64	4,664	15.04 ± 0.03	12.5	12.5	13.5	14.5	16.5	17.5	18.5
1965–69	4,433	14.49 ± 0.03	12.5	12.5	13.5	14.5	15.5	16.5	17.5
1970–74	4,784	14.07 ± 0.03	11.5	12.5	13.5	13.5	14.5	15.5	17.5
1975–79	4,173	13.74 ± 0.04	11.5	11.5	12.5	13.5	14.5	15.5	16.5
1980–84	3,739	13.44 ± 0.03	10.5	11.5	12.5	13.5	14.5	15.5	16.5
1985–89	2,450	13.30 ± 0.04	10.5	11.5	12.5	13.5	14.5	15.5	16.5
1990–94	2,638	13.09 ± 0.04	10.5	11.5	12.5	12.5	13.5	14.5	16.5
1995–99	2,456	12.65 ± 0.03	10.5	11.5	11.5	12.5	13.5	14.5	15.5
2000–04	1,339	12.45 ± 0.04	10.5	11.5	11.5	12.5	13.5	13.5	14.5

SE, standard error.

### 3.2 Trends in age at menarche according to the percentile group

**Figure 2:**
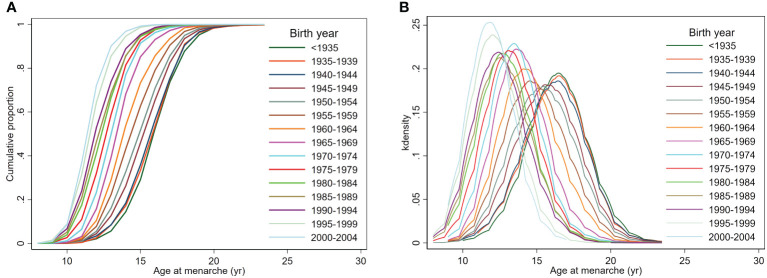
Cumulative proportion and distribution of age at menarche by birth-year. **(A)** The cumulative proportion of reaching menarche at each birth-year, **(B)** the distribution of age at menarche.

From those born before 1935 to those born in 2000–2004, the degree of decline in age at menarche differed according to the percentile group of age at menarche: from 16.5 to 12.5 years in the 50th percentile group, from 13.5 to 10.5 years in the 3rd percentile group (indicative of early menarche), and from 20.5 to 14.5 years in the 97th percentile group (indicative of late menarche) (**Table 1**). The cumulative proportion of menarche by birth-year showed a pattern of decline in age at menarche, and the difference between participants decreased as the birth-year approached the present (**Figure 2A**). For females born more recently, an overall leftward shift toward earlier menarche was observed with a gradual narrowing in the range of the distribution of age at menarche, leading to a reduction in the difference between participants corresponding to the 3rd and 97th percentiles (**Figure 2B**).

### 3.3 Changes of age at menarche based on the percentile group and obesity status

In total, mean age at menarche decreased by –0.071 years per birth-year increment (95% confidence interval [CI], –0.072 to –0.070), with variation across the percentile group of age at menarche; –0.050 years per year (95% CI, –0.052 to –0.048) in the 3rd percentile group, –0.073 years per year (95% CI, –0.074 to –0.072) in the 50th percentile group, and –0.088 years per year (95% CI, –0.091 to –0.085) in the 97th percentile group (*p* <.001 for all). According to obesity status, a more prominent decreasing trend of age at menarche was observed in the obesity group (–0.080 years per year, 95% CI, –0.082 to –0.078) than in the non-obesity group (–0.069 years per year, 95% CI, –0.071 to –0.068) (*p* <.001 for both) (**Table 2**).

**Table 2:** Changes in age at menarche based on the percentile group and obesity status.

	Total	Non-obesity	Obesity
Mean (OLS)	–0.071 (–0.072, –0.070)	–0.069 (–0.071, –0.068)	–0.080 (–0.082, –0.078)
Percentile group of age at menarche			
3rd	–0.050 (–0.052, –0.048)	–0.049 (–0.051, –0.047)	–0.059 (–0.063, –0.055)
10th	–0.059 (–0.060, –0.057)	–0.056 (–0.057, –0.054)	–0.063 (–0.065, –0.060)
25th	–0.067 (–0.067, –0.066)	–0.065 (–0.066, –0.063)	–0.077 (–0.079, –0.075)
50th	–0.073 (–0.074, –0.072)	–0.070 (–0.071, –0.069)	–0.080 (–0.082, –0.078)
75th	–0.082 (–0.083, –0.080)	–0.079 (–0.081, –0.077)	–0.091 (–0.093, –0.089)
90th	–0.085 (–0.087, –0.083)	–0.079 (–0.081, –0.077)	–0.091 (–0.095, –0.087)
97th	–0.088 (–0.091, –0.085)	–0.079 (–0.083, –0.075)	–0.094 (–0.100, –0.088)

OLS, ordinary least squares. Data was expressed as coefficient (95% confidence interval).

## 4 Discussion

Based on the KNHANES 2007–2021 data, we demonstrated an ongoing trend toward an earlier menarche among 50,370 Korean females. The mean age at menarche decreased progressively with birth-year, from 16.92 ± 0.06 years to 12.45 ± 0.04 years in females born between 2000 and 2004 compared to those born before 1935 (–0.071 years per birth-year increment). Stratified analysis according to the percentiles of age at menarche revealed a uniform decline across all percentiles, with a sharper decrease in the late menarche group (97th percentile) than in the early menarche group (3rd percentile). This trend was more pronounced in the obesity group than in the non-obesity group.

The age at menarche significantly influences women’s health outcomes across their lifespan. Early menarche may correlate with precocious puberty during adolescence, potentially leading to a reduced final adult height and triggering psychosocial issues (30, 31). Additionally, an earlier onset of menarche is associated with heightened risks of metabolic syndrome, including impaired glucose tolerance and type 2 diabetes (32, 33). Moreover, females who experience menarche at a younger age have higher incidence of breast and endometrial cancer (1, 34). Furthermore, preliminary evidence suggests that early menarche may contribute to an increased mortality rate from all causes (2).

In Western countries, the age at menarche has significantly decreased from approximately 15.0–17.0 years in the early 1800s to 13.0–13.5 years by the 1950s (13, 35). After the 1950s, this pattern varied among countries. A Dutch study demonstrated a decrease in the median age at menarche from 13.66 years in 1955 to 13.05 years (95% CI, 12.90–13.18) in 2009 (17). A global consortium study indicated a linear decrease in the mean age at menarche across birth-years, from 13.5 years (95% CI, 13.0–14.0) for females born before 1930 to 12.6 years (95% CI, 12.3–13.1) for those born between 1970 and 1984 (16). In Asia, a continuous downward trend of age at menarche has been reported in Chinese females born between 1973 (14.25 years) and 2004 (12.60 years), and in Japanese females born between 1930 (13.8± 1.53 years) and 1985 (12.2 ± 1.39 years) (20, 21). Since the 1980s, a slowdown in this decreasing trend has been observed in some Western countries, such as the USA and Italy (6, 18). Meanwhile, in Norway, the mean age at menarche stabilized from 13.42 years (95% CI, 13.40–13.44) for females born in 1936–1939 to 13.18 years (95% CI, 13.17–13.19) for those born in 1955–1959, then slightly rebounded to 13.24 years (95% CI, 13.22–13.25) for those born in 1960–1964, showing a weak U-shaped pattern (19).

In Korea, previous research using KNHANES 2007–2009 on 11,065 females aged 15 years or older reported that the mean age at menarche had decreased by 0.726 years over a decade, from 13.11 ± 1.52 years in females born between 1980 and 1984 to 12.60 ± 1.14 years in those born between 1990 and 1994 (22). A more recent study using the 2006–2015 Korea Youth Risk Behavior Survey data on 351,006 females aged 12–18 years revealed an ongoing downward trend in age at menarche, from 13.0 years (95% CI, 12.92–13.04) for females born in 1988 to 12.6 years (95% CI, 12.54–12.61) for those born in 2003 (23). In alignment with previous studies, our study using data from the KNHANES 2007–2021 revealed a persistent decreasing trend toward earlier menarche, at a rate of 0.730 years per decade among Korean females born until 2004. Discrepancies in the trend of age at menarche between countries may stem from variations in ethnicity, body habitus, and socioeconomic circumstances (36). Beyond biological and genetic factors, environmental exposure, diet, and lifestyle can also influence the age at menarche (16). In this study, the rate of decline in the age at menarche was faster in the 97th percentile group compared to the 3rd percentile group. This suggests the possibility of a greater contribution of the late menarche group to the ongoing downward trend in age at menarche; but factors driving this phenomenon are yet to be identified.

The global trend toward an earlier onset of puberty over the past half-century is thought to be significantly influenced by improvements in nutritional status and general health, mainly reflected in the increase in childhood BMI (12). Likewise, dose-response relationships have been reported between higher BMI during childhood and the increased likelihood of early menarche (9, 14). Age at menarche was inversely correlated with overweight or obesity (odds ratio, 0.84) among female high school students in Kuwait (37). A study in White-European females reported that an increase in BMI by 1.0 kg/m^2^ at the age of 8 years reduces the age at menarche by 0.26 years, and later menarche was correlated with lower odds of childhood obesity (odds ratio, 0.57) (38). In a Danish cohort study, an increase of 5 kg/m^2^ in BMI was associated with menarche occurring 7.9 months earlier (95% CI, –9.0, –6.6) (10). Consistent with previous studies, our study revealed that menarche tended to begin earlier and faster in females with obesity than in those without obesity, underscoring a possible link between obesity and the timing of menarche. The interaction between leptin, the kisspeptin system, and the peripheral actions of adipose tissue through other adipokines and aromatase activity have been suggested as possible factors influencing the timing of puberty onset (39–41). Additional potential factors accelerating age at menarche may include increased exposure to endocrine-disrupting chemicals that mimic, antagonize, or interfere with the action of endogenous sex steroids, or possess obesogenic properties (42, 43).

Nonetheless, the causal relationship between early menarche and obesity remains uncertain. We observed a similar decreasing trend in the age of menarche among non-obese groups, albeit with a lower slope, suggesting that obesity may not necessarily be the cause of the early menarche. Emerging evidence suggests that early menarche can also be associated with obesity in adulthood. Specifically, early menarche was associated with increased risk of obesity in adulthood (odds ratio, 1.68) and higher BMI in adulthood, with a standardized mean difference of 0.34 kg/m^2^ (3, 32). Further longitudinal studies are necessary to elucidate the causal relationship between early menarche and obesity.

The main strength of our study is that we elucidated the ongoing decreasing trend in age at menarche in recent decades using large nationally representative data. We also identified differences in the trends according to the percentiles of age at menarche and obesity status. Through this approach, our study provides an in-depth insight into the phenomenon of age at menarche. However, our study had several limitations. First, there was a potential possibility of recall bias associated with self-reported age at menarche. Previous research has suggested that a high correlation between recalled and actual age at menarche minimizes this concern (44). Second, we could not assess BMI at the time of menarche due to the retrospective design of the KNHANES data. Nevertheless, studies indicate that childhood obesity serves as an early predictor of obesity in adulthood (28, 29). Finally, the cross-sectional design of our study precluded the establishment of a causal relationship between the age at menarche and obesity.

## 5 Conclusion

Among Korean females born between 1927 and 2004, we identified an ongoing secular trend of an earlier age at menarche, decreasing by 0.710 years per decade. This decline was particularly pronounced among individuals in the higher percentile of the menarche age and those classified as obese. Considering the potential adverse effects of earlier menarche on women’s health, it is crucial to continually monitor the trend of age at menarche and its impact on health outcomes in later life.

## Data availability statement

Publicly available datasets were analyzed in this study. This data can be found here: https://knhanes.kdca.go.kr/knhanes/main.do.

## Ethics statement

The studies involving humans were approved by The IRB of Seoul National University Bundang Hospital (IRB No. X-2403-891-901). The studies were conducted in accordance with the local legislation and institutional requirements. The human samples used in this study were acquired from The KNHANES data is a cross-sectional, nationally representative survey that is conducted annually by the Division of Chronic Disease Surveillance, Korean Centers for Disease Control and Prevention (KCDC). Written informed consent for participation was not required from the participants or the participants’ legal guardians/next of kin in accordance with the national legislation and institutional requirements.

## Author contributions

DHL: Formal Analysis, Writing – original draft. JK: Conceptualization, Data curation, Formal Analysis, Methodology, Writing – review & editing. HYK: Supervision, Validation, Writing – review & editing.

## References

[1] Collaborative Group on Hormonal Factors in Breast C. Menarche, menopause, and breast cancer risk: individual participant meta-analysis, including 118 964 women with breast cancer from 117 epidemiological studies. Lancet Oncol. (2012) 13:1141–51. doi: 10.1016/S1470-2045(12)70425-4 PMC3488186 23084519

[2] Charalampopoulos D McLoughlin A Elks CE Ong KK. Age at menarche and risks of all-cause and cardiovascular death: a systematic review and meta-analysis. Am J Epidemiol. (2014) 180:29–40. doi: 10.1093/aje/kwu113 24920784 PMC4070937

[3] Lee JS Lee YA Shin CH Suh DI Lee YJ Yon DK. Long-term health outcomes of early menarche in women: an umbrella review. QJM. (2022) 115:837–47. doi: 10.1093/qjmed/hcac187 35929081

[4] Prince C Joinson C Kwong ASF Fraser A Heron J. The relationship between timing of onset of menarche and depressive symptoms from adolescence to adulthood. Epidemiol Psychiatr Sci. (2023) 32:e60. doi: 10.1017/S2045796023000707 37766510 PMC10539742

[5] Behboudi-Gandevan S Moe CF Skjesol I Arntzen EC Bidhendi-Yarandi R. The J shaped association of age at menarche and cardiovascular events: systematic review and meta-analysis. Sci Rep. (2024) 14:2695. doi: 10.1038/s41598-024-53011-5 38302648 PMC10834967

[6] Parent AS Teilmann G Juul A Skakkebaek NE Toppari J Bourguignon JP. The timing of normal puberty and the age limits of sexual precocity: variations around the world, secular trends, and changes after migration. Endocr Rev. (2003) 24:668–93. doi: 10.1210/er.2002-0019 14570750

[7] Parent AS Franssen D Fudvoye J Gerard A Bourguignon JP. Developmental variations in environmental influences including endocrine disruptors on pubertal timing and neuroendocrine control: Revision of human observations and mechanistic insight from rodents. Front Neuroendocrinol. (2015) 38:12–36. doi: 10.1016/j.yfrne.2014.12.004 25592640

[8] Gavela-Perez T Garces C Navarro-Sanchez P Lopez Villanueva L Soriano-Guillen L. Earlier menarcheal age in Spanish girls is related with an increase in body mass index between pre-pubertal school age and adolescence. Pediatr Obes. (2015) 10:410–5. doi: 10.1111/ijpo.277 25641777

[9] Juul F Chang VW Brar P Parekh N. Birth weight, early life weight gain and age at menarche: a systematic review of longitudinal studies. Obes Rev. (2017) 18:1272–88. doi: 10.1111/obr.12587 28872224

[10] Brix N Ernst A Lauridsen LLB Parner ET Arah OA Olsen J. Childhood overweight and obesity and timing of puberty in boys and girls: cohort and sibling-matched analyses. Int J Epidemiol. (2020) 49:834–44. doi: 10.1093/ije/dyaa056 PMC7394964 32372073

[11] Kim SJ Kim JH Hong YH Chung IH Lee EB Kang E. 2022 Clinical practice guidelines for central precocious puberty of Korean children and adolescents. Ann Pediatr Endocrinol Metab. (2023) 28:168–77. doi: 10.6065/apem.2346168.084 PMC10556443 37798893

[12] Abreu AP Kaiser UB. Pubertal development and regulation. Lancet Diabetes Endocrinol. (2016) 4:254–64. doi: 10.1016/S2213-8587(15)00418-0 PMC5192018 26852256

[13] Wyshak G Frisch RE. Evidence for a secular trend in age of menarche. N Engl J Med. (1982) 306:1033–5. doi: 10.1056/NEJM198204293061707 7062994

[14] Euling SY Herman-Giddens ME Lee PA Selevan SG Juul A Sorensen TI. Examination of US puberty-timing data from 1940 to 1994 for secular trends: panel findings. Pediatrics. (2008) 121 Suppl 3:S172–91. doi: 10.1542/peds.2007-1813D 18245511

[15] Anderson SE Must A. Interpreting the continued decline in the average age at menarche: results from two nationally representative surveys of U.S. girls studied 10 years apart. J Pediatr. (2005) 147:753–60. doi: 10.1016/j.jpeds.2005.07.016 16356426

[16] Inter LST. Variations in reproductive events across life: a pooled analysis of data from 505 147 women across 10 countries. Hum Reprod. (2019) 34:881–93. doi: 10.1093/humrep/dez015 PMC7571491 30835788

[17] Talma H Schonbeck Y van Dommelen P Bakker B van Buuren S Hirasing RA. Trends in menarcheal age between 1955 and 2009 in the Netherlands. PLoS One. (2013) 8:e60056. doi: 10.1371/journal.pone.0060056 23579990 PMC3620272

[18] Rigon F Bianchin L Bernasconi S Bona G Bozzola M Buzi F. Update on age at menarche in Italy: toward the leveling off of the secular trend. J Adolesc Health. (2010) 46:238–44. doi: 10.1016/j.jadohealth.2009.07.009 20159500

[19] Gottschalk MS Eskild A Hofvind S Gran JM Bjelland EK. Temporal trends in age at menarche and age at menopause: a population study of 312 656 women in Norway. Hum Reprod. (2020) 35:464–71. doi: 10.1093/humrep/dez288 PMC7048709 31990353

[20] Meng X Li S Duan W Sun Y Jia C. Secular trend of age at menarche in chinese adolescents born from 1973 to 2004. Pediatrics. (2017) 140:1–9. doi: 10.1542/peds.2017-0085 PMC5527668 28716824

[21] Hosokawa M Imazeki S Mizunuma H Kubota T Hayashi K. Secular trends in age at menarche and time to establish regular menstrual cycling in Japanese women born between 1930 and 1985. BMC Womens Health. (2012) 12:19. doi: 10.1186/1472-6874-12-19 22800445 PMC3434095

[22] Ahn JH Lim SW Song BS Seo J Lee JA Kim DH. Age at menarche in the Korean female: secular trends and relationship to adulthood body mass index. Ann Pediatr Endocrinol Metab. (2013) 18:60–4. doi: 10.6065/apem.2013.18.2.60 PMC4027094 24904853

[23] Seo MY Kim SH Juul A Park MJ. Trend of menarcheal age among korean girls. J Korean Med Sci. (2020) 35:e406. doi: 10.3346/jkms.2020.35.e406 33350182 PMC7752255

[24] Kweon S Kim Y Jang MJ Kim Y Kim K Choi S. Data resource profile: the Korea National Health and Nutrition Examination Survey (KNHANES). Int J Epidemiol. (2014) 43:69–77. doi: 10.1093/ije/dyt228 24585853 PMC3937975

[25] World Health Organization Regional Office for the Western P. The Asia-Pacific perspective : redefining obesity and its treatment Vol. 2000. . Sydney: Health Communications Australia (2000).

[26] Kim JH Yun S Hwang SS Shim JO Chae HW Lee YJ. The 2017 Korean National Growth Charts for children and adolescents: development, improvement, and prospects. Korean J Pediatr. (2018) 61:135–49. doi: 10.3345/kjp.2018.61.5.135 PMC5976563 29853938

[27] Kim KK Haam JH Kim BT Kim EM Park JH Rhee SY. Evaluation and treatment of obesity and its comorbidities: 2022 update of clinical practice guidelines for obesity by the korean society for the study of obesity. J Obes Metab Syndr. (2023) 32:1–24. doi: 10.7570/jomes23016 36945077 PMC10088549

[28] Simmonds M Llewellyn A Owen CG Woolacott N. Predicting adult obesity from childhood obesity: a systematic review and meta-analysis. Obes Rev. (2016) 17:95–107. doi: 10.1111/obr.12334 26696565

[29] Ward ZJ Long MW Resch SC Giles CM Cradock AL Gortmaker SL. Simulation of growth trajectories of childhood obesity into adulthood. N Engl J Med. (2017) 377:2145–53. doi: 10.1056/NEJMoa1703860 PMC9036858 29171811

[30] Kang S Kim YM Lee JA Kim DH Lim JS. Early menarche is a risk factor for short stature in young Korean females: An Epidemiologic Study. J Clin Res Pediatr Endocrinol. (2019) 11:234–9. doi: 10.4274/jcrpe.galenos.2018.2018.0274 PMC6745461 30604602

[31] Sequeira ME Lewis SJ Bonilla C Smith GD Joinson C. Association of timing of menarche with depressive symptoms and depression in adolescence: Mendelian randomisation study. Br J Psychiatry. (2017) 210:39–46. doi: 10.1192/bjp.bp.115.168617 27491534 PMC5209630

[32] Prentice P Viner RM. Pubertal timing and adult obesity and cardiometabolic risk in women and men: a systematic review and meta-analysis. Int J Obes (Lond). (2013) 37:1036–43. doi: 10.1038/ijo.2012.177 23164700

[33] Cheng TS Day FR Lakshman R Ong KK. Association of puberty timing with type 2 diabetes: A systematic review and meta-analysis. PLoS Med. (2020) 17:e1003017. doi: 10.1371/journal.pmed.1003017 31905226 PMC6944335

[34] Gong TT Wang YL Ma XX. Age at menarche and endometrial cancer risk: a dose-response meta-analysis of prospective studies. Sci Rep. (2015) 5:14051. doi: 10.1038/srep14051 26360785 PMC4566123

[35] Rosenberg M. Menarcheal age for Norwegian women born 1830-1960. Ann Hum Biol. (1991) 18:207–19. doi: 10.1080/03014469100001532 1877808

[36] dos Santos Silva I Beral V. Socioeconomic differences in reproductive behaviour. IARC Sci Publ. (1997) 138:285–308. 9353670

[37] Al-Awadhi N Al-Kandari N Al-Hasan T Almurjan D Ali S Al-Taiar A. Age at menarche and its relationship to body mass index among adolescent girls in Kuwait. BMC Public Health. (2013) 13:29. doi: 10.1186/1471-2458-13-29 23311596 PMC3552970

[38] Bell JA Carslake D Wade KH Richmond RC Langdon RJ Vincent EE. Influence of puberty timing on adiposity and cardiometabolic traits: A Mendelian randomisation study. PLoS Med. (2018) 15:e1002641. doi: 10.1371/journal.pmed.1002641 30153260 PMC6112630

[39] Ahima RS Saper CB Flier JS Elmquist JK. Leptin regulation of neuroendocrine systems. Front Neuroendocrinol. (2000) 21:263–307. doi: 10.1006/frne.2000.0197 10882542

[40] Reinehr T Roth CL. Is there a causal relationship between obesity and puberty? Lancet Child Adolesc Health. (2019) 3:44–54. doi: 10.1016/S2352-4642(18)30306-7 30446301

[41] Mills EG Izzi-Engbeaya C Abbara A Comninos AN Dhillo WS. Functions of galanin, spexin and kisspeptin in metabolism, mood and behaviour. Nat Rev Endocrinol. (2021) 17:97–113. doi: 10.1038/s41574-020-00438-1 33273729

[42] Ouyang F Perry MJ Venners SA Chen C Wang B Yang F. age at menarche, and abnormal menstrual cycle length. Occup Environ Med. (2005) 62:878–84. doi: 10.1136/oem.2005.020248 PMC1740929 16299097

[43] Lopez-Rodriguez D Franssen D Bakker J Lomniczi A Parent AS. Cellular and molecular features of EDC exposure: consequences for the GnRH network. Nat Rev Endocrinol. (2021) 17:83–96. doi: 10.1038/s41574-020-00436-3 33288917

[44] Biro FM Pajak A Wolff MS Pinney SM Windham GC Galvez MP. Age of menarche in a longitudinal US cohort. J Pediatr Adolesc Gynecol. (2018) 31:339–45. doi: 10.1016/j.jpag.2018.05.002 PMC6121217 29758276

