# Supplementary material for: Temporal trend of age at menarche in Korean females born between 1927 and 2004: a population-based study
Source: Front Endocrinol (Lausanne). 2024 Jun 4;15:1399984. doi: 10.3389/fendo.2024.1399984 (PMC11182987; doi:10.3389/fendo.2024.1399984)
Supplement: Supplementary file 1 [file Table_1.docx]

**Supplementary Table 1. Socio-demographic characteristics of the study population**

|  |  | **Birth-year** | | | | | | | | | | | | | | |
| --- | --- | --- | --- | --- | --- | --- | --- | --- | --- | --- | --- | --- | --- | --- | --- | --- |
|  | **Total** | **<1935** | **1935–39** | **1940–44** | **1945–49** | **1950–54** | **1955–59** | **1960–64** | **1965–69** | **1970–74** | **1975–79** | **1980–84** | **1985–89** | **1990–94** | **1995–99** | **2000–04** |
| N | 50730 | 1534 | 2717 | 3506 | 3623 | 3826 | 4848 | 4664 | 4433 | 4784 | 4173 | 3739 | 2450 | 2638 | 2456 | 1339 |
| Age at assessment (years) |  | 78.6  ± 0.1 | 76.2  ± 0.1 | 72.7  ± 0.1 | 67.0  ± 0.1 | 62.2  ± 0.1 | 57.2  ± 0.1 | 52.4  ± 0.1 | 47.4  ± 0.1 | 42.3  ± 0.1 | 37.3  ± 0.1 | 32.6  ± 0.1 | 27.4  ± 0.1 | 22.5  ± 0.1 | 18.4  ± 0.1 | 15.8  ± 0.1 |
| BMI * |  |  |  |  |  |  |  |  |  |  |  |  |  |  |  |  |
| Non-obesity | 35024 (71) | 1031 (69) | 1618 (60) | 1997 (58) | 2075 (58) | 2281 (60) | 3114 (65) | 3234 (70) | 3141 (72) | 3622 (77) | 3261 (79) | 2921 (80) | 1978 (83) | 1744 (84) | 1819 (86) | 1188 (89) |
| Obesity | 14145 (29) | 463 (31) | 1060 (40) | 1442 (42) | 1502 (42) | 1503 (40) | 1701 (35) | 1383 (30) | 1243 (28) | 1106 (23) | 844 (21) | 751 (20) | 393 (17) | 323 (16) | 285 (14) | 146 (11) |
| Residential area | |  |  |  |  |  |  |  |  |  |  |  |  |  |  |  |
| Urban | 40507 (80) | 886 (58) | 1697 (62) | 2403 (69) | 2663 (74) | 2899 (76) | 3777 (78) | 3802 (82) | 3680 (83) | 4120 (86) | 3602 (86) | 3221 (86) | 2165 (88) | 2336 (89) | 2104 (86) | 1152 (86) |
| Rural | 10223 (20) | 648 (42) | 1020 (38) | 1103 (31) | 960 (26) | 927 (24) | 1071 (22) | 862 (18) | 753 (17) | 664 (14) | 571 (14) | 518 (14) | 285 (12) | 302 (11) | 352 (14) | 187 (14) |
| Household income | |  |  |  |  |  |  |  |  |  |  |  |  |  |  |  |
| Low | 10202 (20) | 935 (61) | 1744 (64) | 1924 (55) | 1476 (41) | 928 (24) | 714 (15) | 428  (9) | 380  (9) | 304  (6) | 255  (6) | 195  (5) | 196  (8) | 298 (11) | 297 (12) | 128 (10) |
| Lower-middle | 9737 (19) | 188 (12) | 422 (16) | 758 (22) | 950 (26) | 1039 (27) | 1074 (22) | 786 (17) | 725 (16) | 827 (17) | 761 (18) | 651 (17) | 417 (17) | 473 (18) | 440 (18) | 226 (17) |
| Middle | 9908 (20) | 134 (9) | 209 (8) | 345 (10) | 527 (15) | 738 (19) | 990 (20) | 932 (20) | 904 (20) | 1080 (23) | 1081 (26) | 998 (27) | 566 (23) | 545 (21) | 553 (23) | 306 (23) |
| Upper-middle | 9907 (20) | 103 (7) | 137 (5) | 232 (7) | 354 (10) | 567 (15) | 950 (20) | 1037 (22) | 1073 (24) | 1267 (26) | 1117 (27) | 995 (27) | 576 (24) | 598 (23) | 531 (22) | 370 (28) |
| High | 10450 (21) | 113 (7) | 154 (6) | 192 (5) | 283 (8) | 525 (14) | 1064 (22) | 1442 (31) | 1321 (30) | 1270 (27) | 937 (22) | 872 (23) | 670 (27) | 689 (26) | 613 (25) | 305 (23) |
| Educational level** | | |  |  |  |  |  |  |  |  |  |  |  |  |  |  |
| Elementary school | 13220 (28) | 1386 (90) | 2347 (86) | 2775 (79) | 2346 (65) | 1938 (51) | 1503 (31) | 653 (14) | 152  (3) | 51  (1) | 20  (0) | 19  (1) | 23  (1) | 6  (0) | 1  (0) | 0  (0) |
| Middle school | 4777 (10) | 57  (4) | 140  (5) | 341 (10) | 568 (16) | 779 (20) | 1219 (25) | 931 (20) | 375  (8) | 141  (3) | 65  (2) | 80  (2) | 51  (2) | 22  (1) | 7  (1) | 1  (1) |
| High school | 14438 (31) | 39  (3) | 158  (6) | 268  (8) | 476 (13) | 780 (20) | 1502 (31) | 2068 (44) | 2321 (52) | 2329 (49) | 1481 (35) | 1025 (27) | 728 (31) | 629 (38) | 538 (64) | 96 (93) |
| College | 13885 (30) | 10  (1) | 53  (2) | 92  (3) | 198  (5) | 294  (8) | 590 (12) | 966 (21) | 1526 (34) | 2213 (46) | 2555 (61) | 2553 (68) | 1536 (65) | 995 (60) | 298 (35) | 6  (6) |

Age at assessment is represented as mean ± standard error and number (%). * Missing for 1,561 participants. ** Education level indicates that it is only shown for participants aged 19 and older (n=46,826).

**Supplementary Table 2. Trends in age at menarche among Korean females born between 1927 and 2004 according to the obesity status**

|  |  |  | **Percentile group of age at menarche** | | | | | | |
| --- | --- | --- | --- | --- | --- | --- | --- | --- | --- |
| **Birth year** | **N** | **Mean ± SE** | **3rd** | **10th** | **25th** | **50th** | **75th** | **90th** | **97th** |
| **Non-obesity** |  |  |  |  |  |  |  |  |  |
| <1935 | 1,031 | 16.97 ± 0.08 | 13.5 | 14.5 | 15.5 | 17.5 | 18.5 | 19.5 | 20.5 |
| 1935–39 | 1,618 | 16.87 ± 0.06 | 13.5 | 14.5 | 15.5 | 16.5 | 18.5 | 19.5 | 20.5 |
| 1940–44 | 1,997 | 16.78 ± 0.05 | 13.5 | 14.5 | 15.5 | 16.5 | 18.5 | 19.5 | 20.5 |
| 1945–49 | 2,075 | 16.21 ± 0.05 | 12.5 | 13.5 | 14.5 | 16.5 | 17.5 | 18.5 | 19.5 |
| 1950–54 | 2,281 | 15.94 ± 0.05 | 12.5 | 13.5 | 14.5 | 15.5 | 17.5 | 18.5 | 19.5 |
| 1955–59 | 3,114 | 15.43 ± 0.04 | 12.5 | 13.5 | 14.5 | 15.5 | 16.5 | 17.5 | 18.5 |
| 1960–64 | 3,234 | 15.05 ± 0.04 | 12.5 | 13.5 | 13.5 | 14.5 | 16.5 | 17.5 | 18.5 |
| 1965–69 | 3,141 | 14.53 ± 0.03 | 12.5 | 12.5 | 13.5 | 14.5 | 15.5 | 16.5 | 17.5 |
| 1970–74 | 3,622 | 14.11 ± 0.03 | 11.5 | 12.5 | 13.5 | 14.5 | 14.5 | 15.5 | 17.5 |
| 1975–79 | 3,261 | 13.83 ± 0.03 | 11.5 | 12.5 | 12.5 | 13.5 | 14.5 | 15.5 | 16.5 |
| 1980–84 | 2,921 | 13.55 ± 0.04 | 10.5 | 11.5 | 12.5 | 13.5 | 14.5 | 15.5 | 16.5 |
| 1985–89 | 1,978 | 13.42 ± 0.04 | 10.5 | 11.5 | 12.5 | 13.5 | 14.5 | 15.5 | 16.5 |
| 1990–94 | 1,744 | 13.17 ± 0.05 | 10.5 | 11.5 | 12.5 | 12.5 | 14.5 | 15.5 | 16.5 |
| 1995–99 | 1,819 | 12.76 ± 0.04 | 10.5 | 11.5 | 11.5 | 12.5 | 13.5 | 14.5 | 15.5 |
| 2000–04 | 1,188 | 12.48 ± 0.04 | 10.5 | 11.5 | 11.5 | 12.5 | 13.5 | 13.5 | 14.5 |
| **Obesity** |  |  |  |  |  |  |  |  |  |
| <1935 | 463 | 16.80 ± 0.09 | 13.5 | 14.5 | 15.5 | 16.5 | 17.5 | 19.5 | 20.5 |
| 1935–39 | 1,060 | 16.58 ± 0.07 | 13.5 | 14.5 | 15.5 | 16.5 | 17.5 | 18.5 | 20.5 |
| 1940–44 | 1,442 | 16.51 ± 0.06 | 12.5 | 14.5 | 15.5 | 16.5 | 17.5 | 18.5 | 19.5 |
| 1945–49 | 1,502 | 16.23 ± 0.06 | 12.5 | 13.5 | 14.5 | 16.5 | 17.5 | 18.5 | 19.5 |
| 1950–54 | 1,503 | 15.95 ± 0.06 | 12.5 | 13.5 | 14.5 | 15.5 | 17.5 | 18.5 | 19.5 |
| 1955–59 | 1,701 | 15.57 ± 0.06 | 12.5 | 13.5 | 14.5 | 15.5 | 16.5 | 17.5 | 19.5 |
| 1960–64 | 1,383 | 15.02 ± 0.06 | 12.5 | 12.5 | 13.5 | 14.5 | 16.5 | 17.5 | 18.5 |
| 1965–69 | 1,243 | 14.38 ± 0.05 | 12.5 | 12.5 | 13.5 | 14.5 | 15.5 | 16.5 | 17.5 |
| 1970–74 | 1,106 | 13.95 ± 0.06 | 11.5 | 12.5 | 13.5 | 13.5 | 14.5 | 15.5 | 17.5 |
| 1975–79 | 844 | 13.44 ± 0.06 | 11.5 | 11.5 | 12.5 | 13.5 | 14.5 | 15.5 | 16.5 |
| 1980–84 | 751 | 13.04 ± 0.06 | 10.5 | 11.5 | 12.5 | 13.5 | 14.5 | 15.5 | 16.5 |
| 1985–89 | 393 | 12.71 ± 0.10 | 10.5 | 11.5 | 12.5 | 13.5 | 14.5 | 15.5 | 16.5 |
| 1990–94 | 323 | 12.72 ± 0.09 | 10.5 | 11.5 | 12.5 | 12.5 | 13.5 | 14.5 | 16.5 |
| 1995–99 | 285 | 12.34 ± 0.08 | 10.5 | 11.5 | 11.5 | 12.5 | 13.5 | 14.5 | 15.5 |
| 2000–04 | 146 | 12.25 ± 0.09 | 10.5 | 11.5 | 11.5 | 12.5 | 13.5 | 13.5 | 14.5 |

SE, standard error.
